# Interplay between Electrolyte-Gated Organic Field-Effect Transistors and Surfactants: A Surface Aggregation Tool and Protecting Semiconducting Layer

**DOI:** 10.1021/acsami.1c05938

**Published:** 2021-06-22

**Authors:** Qiaoming Zhang, Adrián Tamayo, Francesca Leonardi, Marta Mas-Torrent

**Affiliations:** † School of Physical Science and Technology, Southwest University, 400715 Chongqing, P. R. China; ‡ Institut de Ciència de Materials de Barcelona, ICMAB-CSIC, Bellaterra, 08193 Barcelona, Spain

**Keywords:** electrolyte-gated organic field-effect transistors, surfactant, surface aggregation, protective top layer, long-term stability

## Abstract

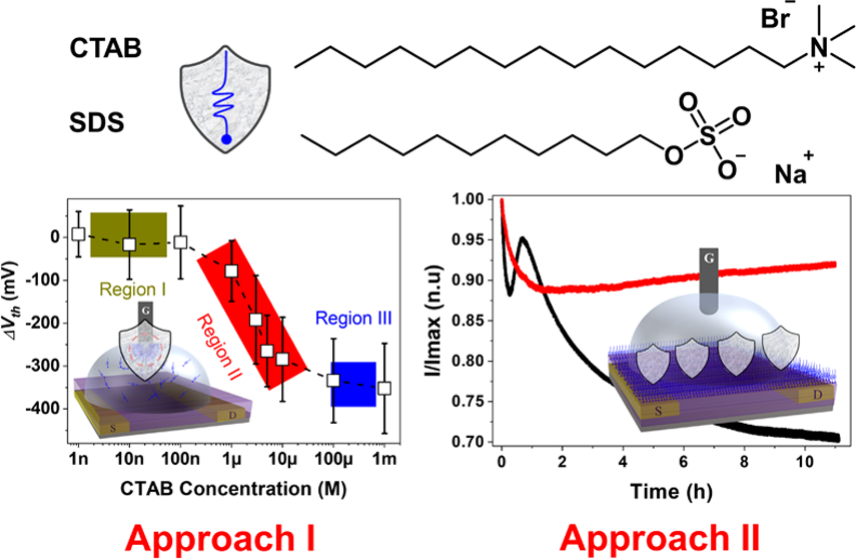

Molecular surfactants, which are based on a water-insoluble tail and a water-soluble head, are widely employed in many areas, such as surface coatings or for drug delivery, thanks to their capability to form micelles in solution or supramolecular structures at the solid/liquid interface. Electrolyte-gated organic field-effect transistors (EGOFETs) are highly sensitive to changes occurring at their electrolyte/gate electrode and electrolyte/organic semiconductor interfaces, and hence, they have been much explored in biosensing due to their inherent amplification properties. Here, we demonstrate that the EGOFETs and surfactants can provide mutual benefits to each other. EGOFETs can be a simple and complementary tool to study the aggregation behavior of cationic and anionic surfactants at low concentrations on a polarized metal surface. In this way, we have monitored the monolayer formation of cationic and anionic surfactants at the water/electrode interface with p*-*type and n*-*type devices, respectively. On the other hand, the operational stability of EGOFETs has been dramatically enhanced, thanks to the formation of a protective layer on top of the organic semiconductor by exposing it to a high concentration of a surfactant solution (above the critical micelle concentration). Stable performances were achieved for more than 10 and 2 h of continuous operation for p*-*type and n-type devices, respectively. Accordingly, this work points not only that EGOFETs can be applied to a wider range of applications beyond biosensing but also that these devices can effectively improve their long-term stability by simply treating them with a suitable surfactant.

## 1 Introduction

Surfactants are amphiphilic molecules that contain a hydrophilic polar head and a long hydrophobic tail.^1−5^ They are widely employed in many areas such as detergents and emulsifiers, in nano- and micro-particle preparation, and even in protein research and DNA extraction.^1,6−8^ The most intriguing property of surfactants is their capability to form supramolecular structures in a solution like micelles and self-assembled structures at the solid/liquid or solid/air interfaces like admicelles, hemimicelles, and monolayers.^1^ The aggregation of surfactants strongly depends on the concentration,^2^ electrolyte ionic strength,^3^ temperature,^9^ and surface properties.^10^ A range of different techniques, including vibrational sum frequency generation (SFG) spectroscopy combined with total internal reflection Raman (TIR Raman) scattering^1^ or with surface plasmon resonance (SPR)^2^ and atomic force microscopy (AFM),^3,11^ are commonly employed to investigate the aggregation of this class of molecules on surfaces. However, these techniques are not easily available and, further, the data interpretation can be complex.

Organic electronic devices are raising considerable interest for applications requiring low cost since organic semiconductors (OSCs) can be printed on flexible substrates over large areas. In particular, electrolyte-gated organic field-effect transistors (EGOFETs) are appealing for working in aqueous media.^12−14^ Their layout consists of exposing directly the OSC toward an electrolyte, where a gate contact is also immersed. The application of a source–gate voltage yields the formation of two electrical double layers (EDLs): one placed at the gate/electrolyte interface and the other one at the OSC/electrolyte interface. These EDLs, which exhibit high capacitance values on the order of tens of μF·cm^–2^, determine the device’s electrical performance.^15^ For this reason, EGOFETs have often been exploited as electrical transducers for biosensing by the proper modification of such interfaces with receptor groups.^16−18^ Additionally, although much less explored, EGOFETs can also be employed as a research tool to study chemical/physical processes or to monitor cell/microorganism activities,^19−21^ where such events can be followed by registering an easily readable electrical signal. However, despite the high potential of these devices, some unsolved problems are hindering their technological transfer such as their long-term electrical stability in aqueous media.

In this work, we demonstrate the reciprocal benefits that surfactants and EGOFETs can offer to each other. First, we show that EGOFETs can become an appealing alternative tool to investigate the adsorption behavior of cationic and anionic surfactants on a polarized metal surface (i.e., gate electrode) by p*-* or n*-*type EGOFETs, respectively. Second, we demonstrate unprecedented long-term operation stability of EGOFETs upon exposing the OSC layer to a high concentration (above the critical micelle concentration, CMC) of a surfactant solution due to the formation of a protective top layer on the OSC. Hence, our current work further evidences the high potential of EGOFETs, proving that these devices can exhibit improved electrical performance and that they can be applied to a broader range of applications.

## 2 Results and Discussion

**Figure 1:**
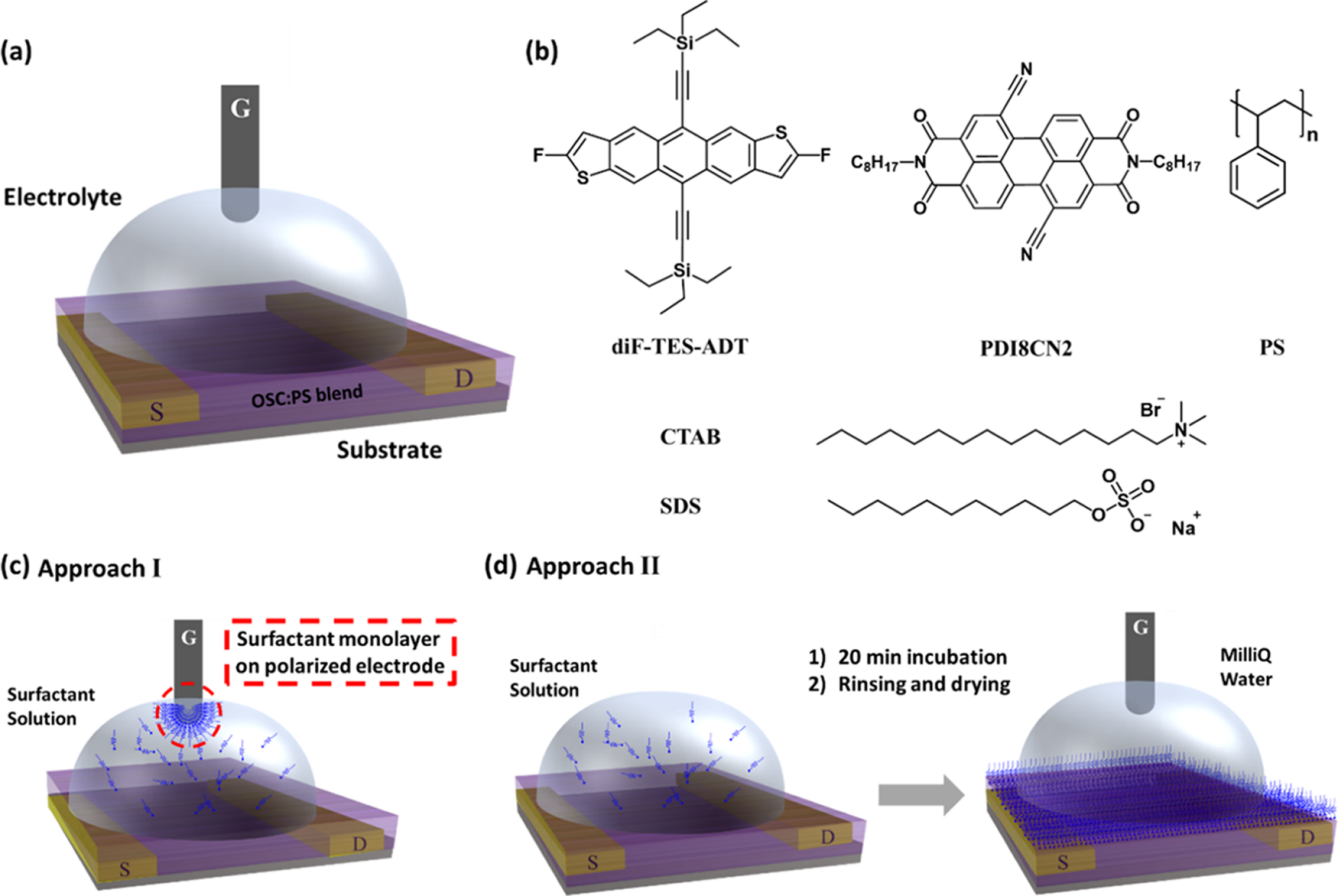
(a) Scheme of the EGOFET structure. (b) Molecular structures of the OSCs, PS, and surfactants used in this work. Schematic detection procedure of (c) approach I and (d) approach II.

The EGOFET architecture is shown in Figure 1a. It consists of an OSC layer bottom-contacted by two gold electrodes, namely, source (S) and drain (D). The OSC is in electric contact with a third Pt gate electrode (G) through an electrolyte. These devices operate at a very low operation voltage (<1 V) due to the high capacitance of electrical double layers. The application of source–gate voltage (*V*_GS_) promotes the formation of two EDLs at the electrolyte/OSC and electrolyte/gate electrode interfaces,^12,15^ where the applied *V*_GS_ decreases steeply.^22,23^ The OSC represents the core element of this device. To obtain devices that are electrically stable in water, highly crystalline and homogeneous OSC thin films are required to avoid electrochemical doping. Previously, we showed that the deposition of blends of OSCs with an insulating polymer (i.e., polystyrene, PS) by the solution shearing technique (i.e., bar-assisted meniscus shearing, BAMS) is an effective strategy to realize high-performance EGOFET devices.^12,15^ This was ascribed to the high degree of thin-film crystallinity along with an extended homogeneity at the long range (i.e., from several to hundreds of micrometers). Therefore, in this work, we have followed the same approach and, as active materials, used PS-based blends of p-type OSC 2,8-difluoro-5,11-bis(triethylsilylethynyl)anthradithiophene (diF-TES-ADT) and n-type OSC *N*,*N*′-bis(*n*-octyl)-dicyanoperylene-3,4:9,10-bis-(dicarboximide) (PDI8CN2). The chemical structures of these materials are shown in Figure 1b (see Section 4 for further details regarding the deposition of the materials and device fabrication).

Regarding surfactants, we selected cationic surfactant cetyltrimethylammonium bromide (CTAB) and anionic surfactant sodium dodecyl sulfate (SDS) (Figure 1b, bottom). Surfactants show different aggregation behaviors according to their concentration. The critical micelle concentration (CMC) refers to the minimum concentration required for micelle formation in solution, which can then assemble in a different fashion once a surface is available. At high surfactant concentrations above CMC, the morphology of surfactant aggregates on the solid surface vary from globular micelles to cylindrical micelles and, finally, to layered films.^11^ At the other extreme, the monolayer formation concentration (MFC), which for a given surfactant is typically several orders of magnitude below its CMC, does not allow the formation of micelles in solution and, at the solid–liquid interface normally promotes the formation of a molecular monolayer.^1,2,4,6^

### 2.1 Approach I: EGOFETs as a Tool to Study the Monolayer Formation on a Polarized Metal Surface

In this part of the work, we aimed at exploiting EGOFETs as an ultrasensitive tool to investigate the monolayer formation of surfactants at the water–metal interface, specifically on the EGOFET polarized Pt gate contact. For this purpose, to monitor the cationic CTAB surfactant on a negatively charged electrode, a p-type EGOFET was required. On the contrary, the anionic SDS was studied by employing an n-type EGOFET.

**Figure 2:**
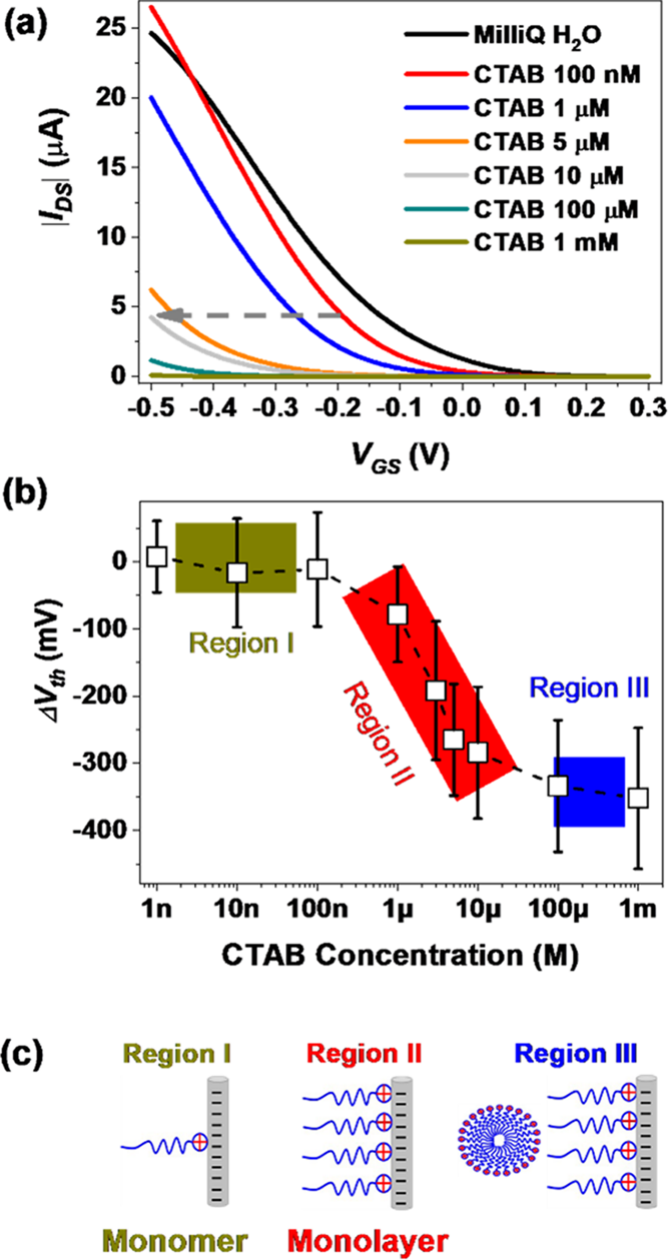
(a) *I*–*V* transfer characteristics of p-type EGOFETs (based on diF-TES-ADT:PS blend) in the saturation regime (*V*_GS_ = −0.4 V) using CTAB solutions in Milli-Q water as the electrolyte. (b) Average threshold voltage shifts (Δ*V*_th_) plotted against different concentrations of CTAB. These data were extracted from five devices. The EGOFET devices were exposed to CTAB solutions with concentrations ranging from 1 nM to 1 mM in ascending order. (c) Schematic picture of CTA^+^ aggregation on the gate Pt surface.

Figure 2a displays the typical p-type transfer characteristics (*V*_GS_ window ranging from 0.3 to −0.5 V) of an EGOFET based on diF-TES-ADT:PS using Milli-Q water as electrolyte media (black curve). A stability check consisting of recording several transfer characteristics was always performed to ensure a reproducible electrical response before the subsequent surfactant detection test (Figure S1a, Supporting Information). Afterward, water solutions with different concentrations of CTAB spanning from 1 nM to 1 mM were directly employed as electrolyte media in ascending order (Figure 1c, approach I). For each concentration, an additional stability check in different CTAB concentrations was carried out to ensure the stability and reproducibility of the data (see, for instance, the device response with 1 μM CTAB in Figure S1b, Supporting Information). Clearly, transfer characteristics are significantly affected by the addition of CTAB in the media, as evidenced by the negative *V*_th_ shift accompanied by a decrease in the source–drain current (*I*_DS_) in the saturation regime (Figure 2a). It is also worth noting that the slopes of transfer curves ( vs *V*_GS_ curves) are not significantly altered in the whole experiment (Figure S1c-d, Supporting Information), indicating that product [mobility × capacitance] (μ × *c*_dl_) is not much influenced (the average value is 0.23 μS·V^–1^ with 21% variation). The same scenario is found when plotting the electrical characteristics in the linear regime (*V*_DS_ = −0.1 V) (Figure S2, Supporting Information). These observations can be rationalized by considering the aggregation behavior of the CTAB cations (hereafter referred to as CTA^+^) on the negatively polarized Pt gate surface. This phenomenon leads to effective screening of the gate bias, which results in a negative shift of *V*_th_, and thus, a higher *V*_GS_ is required to create a conducting channel in the OSC. The same experiment was repeated by replacing CTAB with NaBr to keep the same anion while dramatically changing the cationic counterpart. In this case, a much less pronounced negative *V*_th_ shift was observed (see Figure S3, Supporting Information). This supports our previous assumption regarding the self-organization of the large CTA^+^ cations on the gate electrode, which causes a stronger screening of the *V*_GS_.

To quantify the dependence of *V*_th_ shift with respect to CTAB concentration, *V*_th_ was extracted in linear and saturation regimes (Section 4). The *V*_th_ shift (Δ*V*_th_) is defined as Δ*V*_th_*= V*_th_^CTAB^*– V*_th_^MilliQ water^, where *V*_th_^CTAB^ is the *V*_th_ under different CTAB concentrations and *V*_th_^MilliQ water^ corresponds to the value extracted when Milli-Q water is employed as electrolyte media before the addition of the surfactant. The relationship between Δ*V*_th_ and log[CTAB] is depicted in Figure 2b, where three different regions can be clearly observed: a first plateau region (I) at low surfactant concentrations (1 nM < *c* < 100 nM), a steep region (II) at medium concentrations (100 nM < *c* < 10 μM), and a second plateau (III) at high concentrations (10 μM < *c* < 1 mM).

The CMC of CTAB in water is known to be 1 mM,^24^ whereas its MFC is around 1–10 μM in water.^5^ Taking this into consideration, region (I) can be attributed to the adsorption of monomers on the gate caused by electrostatic interactions between the positively charged head-group of CTA^+^ and the negatively polarized Pt gate electrode, as sketched in Figure 2c. Such monomer aggregation results in negligible effects on the EGOFET response. In region (II), an abrupt *V*_th_ shift due to effective screening of the gate bias is found, which might be indicative of the surfactant monolayer formation on the gate electrode. In region (III), above 10 μM, CTA^+^ ions fully cover the surface of the Pt gate electrode, and then, an increase in surfactant concentration is no further affecting the device’s electrical characteristics.

The influence of the ionic strength was also explored by phosphate-buffered solution (PBS, 1×, pH 7.4) as electrolyte media, giving a similar trend (Figure S4, Supporting Information). This result is also in agreement with the fact that, as expected, CTAB has a head-on orientation of the CTA^+^ ions on the negatively polarized Pt surface. This can be affirmed because the Debye screening length (λ_D_) sharply decreases with increasing ionic strength.^6,25,26^ At high ionic strengths, the CTA^+^ charges would not be detected if they were oriented far away from the Pt gate (i.e., λ_D_ is ∼100 nm in Milli-Q water, while it is <1 nm in PBS 1× solution^27^). A similar response was observed by replacing the Pt gate electrode with a carbon composite-based electrode (Figure S5, Supporting Information). Furthermore, cyclic voltammetry (CV) and electrochemical impedance spectroscopy (EIS) experiments further support the formation of a CTA^+^ monolayer on the Pt electrode (Figures S6 and S7, Supporting Information). Finally, atomic force microscopy (AFM) images on evaporated gold electrodes on Kapton after exposure to CTAB at a concentration of 10 μM (region II) and applying a voltage sweep from 0.3 to −0.5 V also confirmed the formation of a monolayer (Figure S8, Supporting Information). However, the presence of CTAB on the OSC surface was not observed under these conditions (Figure S9, Supporting Information). Noticeably, the influence of CTAB aggregation on the OSC surface was also explored by incubating the film with the surfactant previously to perform the electrical test, but not significant electrical effects were observed in the monolayer formation concentration range (Figure S10, Supporting Information).

It should be mentioned that previously an organic electrochemical transistor (OECT), whose working principle relies on doping/dedoping a conducting polymer, was reported as a suitable platform for the detection of CTAB micelles.^28^ This was driven by the modification of the size and type of ionic dopants occurring during CTAB micelle assembly. However, with the here-in fabricated EGOFETs, we are able to study the CTAB monolayer formation, which takes place at much lower concentrations.

**Figure 3:**
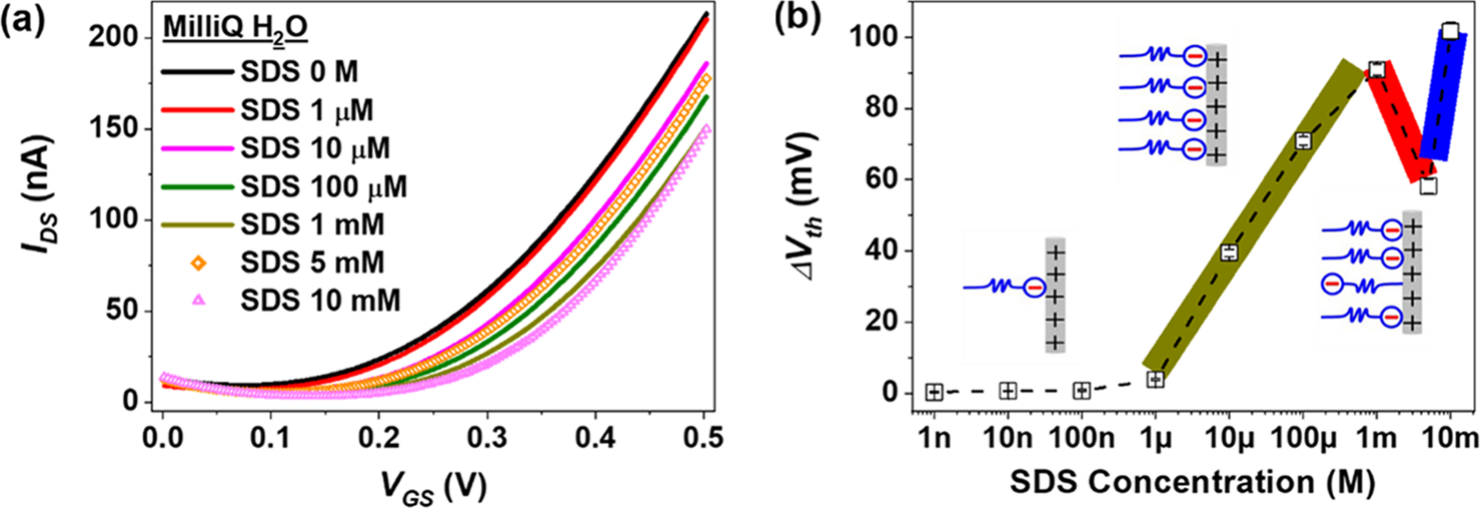
(a) *I*–*V* characteristics in the saturation regime (*V*_DS_ = 0.5 V) of an n-type EGOFET (based on PDI8CN2:PS blend) using SDS solutions of different concentrations in Milli-Q water as electrolyte media. (b) Δ*V*_th_ vs log[SDS] extracted from three different devices. The n-type EGOFET device was exposed to SDS surfactant solutions from 1 nM to 10 mM in ascending order.

To verify the versatility of this approach, the surface assembly of anionic surfactant SDS was investigated using an n-type EGOFET based on a PDI8CN2:PS blend film.^29^ In this device, the Pt gate electrode is positively polarized during its operation. Figure 3a displays the transfer characteristics in the saturation regime of the device exposed to different SDS concentrations. Noticeably and as the previous example, the electrical characteristics are significantly affected by the presence of the SDS surfactant due to the screening of the gate voltage by the SDS anion (hereafter referred to as DS^–^) assembly on the gate electrode, resulting in a *V*_th_ shift toward higher-voltage values together with a reduction of *I*_DS_ (Figure 3a).

Analyzing in detail the Δ*V*_th_ shift as a function of the SDS concentration (Figure 3b), we observe that the results agree with DS^–^ monolayer formation in the concentration range from 1 μM to 1 mM. However, at higher concentrations, an oscillating trend of the Δ*V*_th_ is found, which seems to be in accordance with the work published by Song and co-workers, wherein SFG and SPR characterization techniques were employed.^2^ They found that a DS^–^ monolayer is formed at a concentration of 0.2 mM by the initial adsorption of DS^–^ molecules with their negatively charged head groups pointing toward the positively polarized surface. Nevertheless, the decrease of surface potential promotes that, at higher SDS concentrations (3 mM < *c* < 8 mM), some SDS molecules begin adsorbing in the opposite orientation (head groups pointing toward the water phase). Such a process would explain the reduction of *V*_GS_ screening observed in our devices at SDS concentrations above 1 mM, as sketched in the inset of Figure 3b. The CMC of SDS is reported to be around 7–8 mM in deionized water;^30^ hence, above this concentration, micelles are formed.

All of these results have further been confirmed by EIS measurements, in which the capacitance response also exhibits an oscillating trend in the same SDS concentration range (Figure S11, Supporting Information). Noticeably, this behavior disappeared when using PBS 1× as a medium (Figure S12, Supporting Information), probably caused by the decrease of λ_D_ at high ionic strengths,^26,31^ which prevents the detection of the head-out orientation of DS^–^ anions. AFM analysis of a planar gold gate electrode after being exposed to SDS at a concentration of 1 mM and applying a transfer voltage sweep also confirmed the presence of a monolayer on the electrode (Figure S13, Supporting Information).

Thus, EGOFETs represent a promising platform to study the surface assembly of ionic surfactants at low concentrations on a polarized electrode using a simple electrical read-out.

### 2.2 Approach II: Surfactant Protecting Layer to Enhance the Long-Term Stability of EGOFETs

Long-term operational instability is a key factor for sensor implementation in biological and biomedical applications.^15^ Stability is still a major drawback in EGOFETs, and it is mainly ascribed to the migration of ions into the OSC layer.^15^ Therefore, finding a suitable route to protect the OSC film, without hampering the electrical performance, could be an effective way to enhance the device’s stability.

It is known that when the concentrations of surfactant solutions are at least 2 times higher than their CMC, micelles aggregate on surfaces, finally forming layered films.^11^ Taking this into account, we proceed in using this approach for encapsulating the OSC films. Approach II consisted hence of placing a drop of the surfactant solution with a concentration above CMC on top of the OSC film for 20 min, followed by the removal of the solution and then drying with a N_2_ flow. Then, the EGOFET was electrically tested in Milli-Q water (Figure 1d).

**Figure 4:**
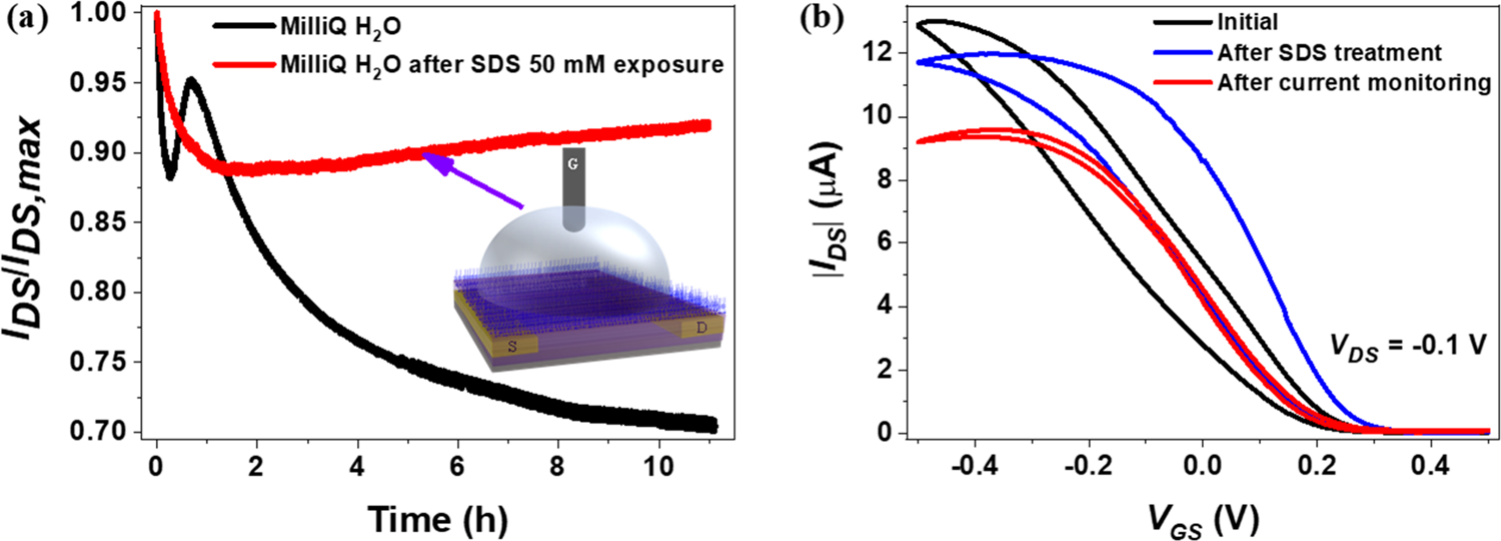
(a) *I*–*t* plot of the normalized *I*_DS_ of a p-type EGOFET (based on diF-TES-ADT:PS blend) recorded at *V*_GS_ = −400 mV and *V*_*DS*_ = −50 mV in the absence of SDS and after treating the organic semiconductor film with SDS. All data were recorded using Milli-Q water as the electrolyte. The inset is the schematic interpretation of the SDS aggregation on the diF-TES-ADT:PS blend surface. (b) *I*–*V* transfer characteristics in the linear regime using Milli-Q water as media recorded initially (black line), after treating the semiconductor with SDS (blue line), and after the current monitoring test of the SDS treated device (red line).

The first test was performed using the p*-*type EGOFET based on diF-TES-ADT:PS and the anionic SDS surfactant. To optimize the SDS concentration, a series of different concentrations of SDS solutions in Milli-Q water were employed. Current monitoring tests under fixed *V*_GS_ = −400 mV and *V*_DS_ = −50 mV were performed to test their protective efficacy. As displayed in Figure S14 (Supporting Information), the optimized SDS concentration was 50 mM. To highlight the long-term stability induced by the SDS layer, the normalized and original current monitoring data obtained from devices with and without the SDS treatment are presented in Figures 4a and S15 (Supporting Information), respectively. In the absence of SDS (black curve), the *I*_DS_ exhibits a fast decrease in the first half-hour followed by a current increase in the following 30 min and then a slow but continuous decrease for the remaining test time (more than 30% of the current is dropped after 10 h). In contrast, the EGOFETs treated with SDS show an initial 10% current decrease but, afterward, show a stable current value for more than 10 h (red curve). This enhanced and unprecedented operation stability is mainly ascribed to the protective action provided by the SDS layer formed on top of the OSC film, as sketched in the inset of Figure 4a. Our observations are further confirmed by the transfer characteristics recorded prior to and after *I*/*t* monitoring. As shown in Figure 4b, in the transfer characteristics registered after the SDS treatment (blue line), only a small positive *V*_th_ shift accompanied with *I*_DS_ increase is found with respect to the initial device (black line). These results are attributed to the DS^–^ anions that aggregate on the OSC surface, inducing more holes in the conduction channel. After the current monitoring test (red line), the device shows a stable electrical performance with a slight *I*_DS_ decrease and a significantly reduced hysteresis. It should be noticed that only when the device is operated at a high source–drain bias (*V*_DS_ = −400 mV), the enhanced device stability is no more realized, which could be attributed to the high longitudinal field present along the OSC channel (Figure S16, Supporting Information).

AFM was employed to study the morphology of the OSC thin-film surface prior to and after exposure to the SDS surfactant solution. As shown in Figure S17 (Supporting Information), the AFM images reveal morphological features at the nanoscale level on top of the diF-TES-ADT:PS thin film, which can be attributed to the SDS assemblies.

Similar results were obtained using films of only diF-TES-ADT (i.e., without the binding PS polymer) or also using another anionic surfactant such as sodium octyl sulfate (SOS) (Figures S18 and S19, Supporting Information). Finally, a comparison experiment was carried out by substituting SDS for the cationic surfactant CTAB following the same experimental protocol (Figure S17, Supporting Information). As displayed in Figure S20 (Supporting Information), no device stability improvement was noticed in this case. This indicates that with the cationic surfactant the protective layer on the OSC is not stably formed under the operational device conditions of a p-type EGOFET, which can be ascribed to the polarization of the device (the OSC is positively charged under operation).

**Figure 5:**
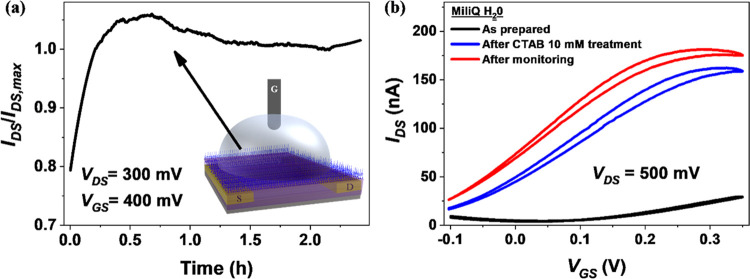
(a) *I*–*t* plot of EGOFETs based on PDI8CN2:PS films. The normalized *I*_DS_ is recorded at fixed operation voltage (*V*_GS_ = 400 mV and *V*_DS_ = 300 mV) using Milli-Q water as the electrolyte and after treating the OSC film with a 10 mM CTAB solution. The inset is the schematic view of CTAB aggregation on the OSC surface. (b) *I*–*V* transfer characteristics recorded in the saturation regime using Milli-Q water as electrolyte media of the device as prepared (black line), after CTAB treatment (blue line), and after the current monitoring test (red line).

To further verify this and validate our methodology, PDI8CN2:PS films were exposed to CTAB (10 mM solution) and implemented as n-type EGOFET. As in the previous case, AFM characterization shows the formation of a passivating layer on top of the organic semiconductor film after exposing it to CTAB (Figure S21, Supporting Information). It is important to highlight that n-type EGOFET devices suffer much fast deterioration due to moisture and water.^32^ As displayed in Figure 5a, the same stability enhancement observed previously with the p*-*type EGOFET was encountered again here. Indeed, a sharp *I*_DS_ increase in the first half-hour followed by a constant *I*_DS_ in the following 2 h was found. In stark contrast, the n-type EGOFET with no protection shows fast and important deterioration with about 90% decrease of the initial current after 3 h (Figure S22, Supporting Information). This behavior is further confirmed by comparing the transfer characteristics prior to and after the current monitoring test (Figures 5b and S22, Supporting Information). After protecting the organic semiconductor with CTAB, an important shift of the transfer characteristics takes place. Remarkably, after 3 h of continuous operation during the monitoring test, the transfer characteristics registered prove that the device is still working properly.

## 3 Conclusions

In conclusion, we have fabricated p-type and n-type EGOFETs based on printed OSC:PS blends and studied the effect of the surface aggregation of surfactants on their interfaces, which is highly influenced by the device polarization. First, the EGOFETs have been demonstrated to be a simple, complimentary, and highly sensitive tool to investigate the adsorption behavior of cationic (i.e., CTAB) and anionic (i.e., SDS) surfactants on the gate surface of EGOFETs. Employing a p*-*type EGOFET, the formation of a monolayer of the CTA^+^ cations on the negatively polarized Pt gate electrode could be monitored. This was evidenced by a sharp *V*_th_ variation in the concentration range (1 μM < *c* < 10 μM). Additionally, with an n-type EGOFET, the surface assembly of SDS was explored. The results are in agreement with a head-on monolayer formation up to a concentration of 1 mM. Afterward, the oscillating trend of *V*_th_ found indicates that some SDS molecules upturn adsorbing on the surface in the opposite orientation.

In the second part of the work, surfactants were exploited to spontaneously form a protective layer for the OSC thin films to prolong EGOFETs’ long-term stability. In particular, forming a layer of the anionic SDS surfactant on top of a p*-*type OSC unprecedentedly enhanced the device’s stability for more than 10 h in continuous operation. Similarly, the cationic CTAB surfactant improved the water stability of an n-type OSC for more than 2 h.

Our results point not only that EGOFETs can be applied to a wider range of applications beyond biosensing but also that these devices can exhibit long-term stability when operating in a water environment by simply treating them with a suitable surfactant. Hence, low-cost EGOFET devices might become a simple and versatile tool for analysis in laboratories covering different fields.

## 4 Experimental Section

### 4.1 Materials

The organic semiconductor materials used in this work, 2,8-difluoro-5,11-bias(triethylsilylethynyl)anthradithiophene (diF-TES-ADT) and *N*,*N*′-bis(*n*-octyl)-dicyanoperylene-3,4:9,10-bis-(dicarboximide) (PDI8CN2), were purchased from Lumtec and Polyera Inc. (N1200), respectively. Polystyrene (PS, *M*_w_ = 10 000 and 280 000 g·mol^–1^), chlorobenzene (CB), and 2,3,4,5,6-pentafluorothiophenol (PFBT) were provided by Sigma-Aldrich. The cetyltrimethylammonium bromide (CTAB) and sodium dodecyl sulfate (SDS) were also purchased from Sigma-Aldrich. All of the commercial materials were used as received without any further purification. Platinum (Pt) wire (Φ =0.5 mm) and silver (Ag) wire (Φ =0.5 mm) were obtained from Sigma-Aldrich. Multiwalled carbon nanotubes (>95% of carbon purity, 10–30 nm of outer diameter, and about 5–15 μm of length) were purchased from SES Research (Houston, TX). Pt wire was cleaned in hot sulfuric acid (0.1 M) for 30 min and rinsed with Milli-Q water prior to each electrical and electrochemical measurement. Milli-Q water (18.2 MΩ·cm resistivity at 25 °C) was obtained from a Milli-Q Integral Water Purification System.

### 4.2 EGOFET Device Fabrication

The diF-TES-ADT:PS_10K_ (4:1) and PDI8CN2:PS_280K_ (1:2) blend solutions used in this work were prepared according to our previously published protocol.^15,29^ The *S*/*D* electrodes (Cr/Au, 5/40 nm), with a geometrical ratio *W*/*L* = 690 (channel width *W* = 20 700 μm and channel length *L* = 30 μm), were deposited on Kapton polyimide film (75 μm thick, DuPont) by a thermal evaporation system (Evaporation System Auto 306 from Boc Edwards) at a pressure of 2 × 10^–4^ Pa. Prior to the deposition of the OSC:PS blend solution, the *S*/*D* substrates were cleaned in an ultrasonic bath with acetone and isopropanol for 15 min each, followed by an ozone treatment for 25 min. After that, the cleaned *S*/*D* electrodes were functionalized by PFBT through immersion of the substrate into PFBT/isopropanol (2 μL:1 mL) solution for 15 min and then rinsed with isopropanol. Finally, all of the OSC:PS blend films were coated by the bar-assisted meniscus shearing (BAMS) technique at a fixed speed of 1 cm·s^–1^ and a plate temperature of 105 °C in ambient conditions as previously reported.^15^

### 4.3 Surfactant Solution Preparation

Surfactant solutions with different concentrations were prepared in Milli-Q water and phosphate-buffered saline (PBS 1×, pH 7.4 with 137 mM NaCl, 2.7 mM KCl, 10 mM Na_2_HPO_4_, 2 mM KH_2_PO_4_). All of the original surfactant solutions were prepared at a concentration of 100 mM by dissolving the corresponding surfactant salts and then stirring at 40 °C for at least 1 h to completely dissolve the powder. Afterward, the solutions were diluted as required with Milli-Q water or PBS solution. All of the surfactant solutions were freshly prepared before each experiment.

### 4.4 Electrical Measurements

Electrical measurements, including transfer, output characteristics, and long-term stability tests, were recorded in ambient conditions using an Agilent B1500A semiconductor device analyzer connected to the samples with a Karl SÜSS probe station. For the surfactant aggregation behavior study (approach I), a poly(dimethylsiloxane) (PDMS) pool was placed on top of the interdigitated area of the EGOFETs to confine 30 μL of target aqueous solution as electrolyte media and a gate electrode (Pt wire, carbon paste electrode), as depicted in Figure 1c. The surfactants, dissolved in Milli-Q water or PBS 1× solution, were employed directly as electrolyte media during the EGOFET tests in a concentration spanning from nanomolar to millimolar. Carbon paste electrode was fabricated according to a previously reported protocol.^33,34^ The scan rate of transfer characteristics was set to ∼70 mV·s^–1^ unless otherwise specified.

To investigate the in situ long-term EGOFET monitoring after the formation of a surfactant protecting layer on top of the OSC (approach II), a drop of surfactant solution (∼50 μL) with target concentrations (1, 5, 10, 50, and 100 mM) was placed on top of the active area and kept for 20 min under ambient conditions to allow the self-assembly of a compact surfactant layer on top of the semiconductor (Figure 1d, left). Then, the solution was removed by gentle rinsing and dried with N_2_. A large PDSM pool (∼500 μL) was fixed on top of the interdigitated area followed by the injection of Milli-Q water into the pool as electrolyte media (Figure 1d, right). During the long-term stability test, a parafilm was sealed on top of the pool to minimize solvent evaporation during the test. *I*_DS_ was continuously recorded with an interval time of 1 s at *V*_GS_ = −400 mV and *V*_DS_ = −50 or −100 mV for p-type devices, whereas for n-type devices, we applied *V*_GS_ = 500 mV and *V*_DS_ = 100 mV.

To quantitatively describe the properties of EGOFETs, the classical MOSFET model has been employed to extract the figure of merits, including the threshold voltage (*V*_th_), total electrical double layers capacitance (*C*_dl_), and charge carrier mobility (μ). According to the MOSFET model, the *I*_DS_ values in linear and saturation regimes are approximately determined by the following equations
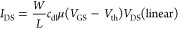

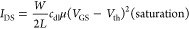
where *W* is the channel width and *L* is the channel length.

### 4.5 Morphological Characterization

Atomic force microscopy (AFM) images were obtained working with a 5100 SPM system from Agilent Technologies in tapping mode. For approach I, gold gate electrodes were evaporated on Kapton and analyzed by AFM after being exposed to ML and CMC concentration of CTAB (10 μM and 10 mM, respectively) and SDS (1 and 50 mM, respectively) while a voltage sweep was applied (from 0.3 to −0.5 V for CTAB and from −0.3 to 0.5 V for SDS) to them using a Novocontrol Alpha-AN impedance analyzer equipped with POT/GAL 30V/2A. To check the formation of the surfactant protective layer on the OSC thin film, the freshly prepared OSC thin films were first investigated. Then, the samples were dipped into a surfactant solution at a concentration above its CMC for 20 min and then slightly rinsed and dried with N_2_ gas. All images were recorded in ambient conditions and analyzed using Gwyddion 2.47 software.

### 4.6 Electrochemical Impedance Spectroscopy (EIS)

Cyclic voltammetry (CV) and electrochemical impedance spectroscopy (EIS), including capacitance and phase angle spectra, were carried out using a Novocontrol Alpha-AN impedance analyzer equipped with a POT/GAL 30V/2A electrochemical interface in a frequency range between 10^5^ and 10^–1^ Hz. CV measurements were carried out in a conventional three-electrode electrochemical cell using a Pt wire as the working electrode (Φ = 0.5 mm), a second Pt wire as the counter electrode (Φ = 0.5 mm), and an Ag/AgCl quasi-reference electrode (Φ = 0.5 mm). Electrochemical responses were recorded in 0.1 M KCl aqueous solution containing 0.1 mM [Fe(CN)_6_]^3–/4–^ as the redox marker. During the CV test, a 100 mM CTAB solution in Milli-Q water was added to the electrochemical cell to achieve the desired concentration of CTAB in the working solution. The scan rate of the CV measurements was set to 10 mV·s^–1^. For the impedance measurements, a square Au electrode (*S* = 1 mm^2^) was employed as the counter electrode, a Pt wire (Φ = 0.5 mm) served as the working electrode, and Ag/AgCl wire (Φ = 0.5 mm) was used as the quasi-reference electrode. Surfactant solutions of different concentrations (in ascending order) were used as media confined with a PDMS pool on the counter electrode. Prior to each EIS test, the CV measurement was implemented to guarantee the migration and adsorption of CTA^+^ or DS^–^ on the Pt surface, which was obtained by defining the DC voltage in the same window (0.3 to −0.5 V, a step of −0.1 V in the CTAB case, while 0 to 0.5 V, a step of 0.1 V in the SDS case) used for the EGOFET transfer characteristics. Then, the surfactant solutions were replaced by Milli-Q water. During EIS tests, DC and AC signals were fixed to 0 and 20 mV, respectively.

## Supporting Information Available

Additional electrical transfer characteristics of EGOFETs, CV and EIS characterizations, AFM images, determination of the optimized surfactant concentration for the formation of a protective layer on top of OSC in p-type EGOFETs, and additional *I*–*t* stability plots (PDF)
