# Supplementary material for: Interplay between Electrolyte-Gated Organic Field-Effect Transistors and Surfactants: A Surface Aggregation Tool and Protecting Semiconducting Layer
Source: ACS Appl Mater Interfaces. 2021 Jun 22;13(26):30902–9. doi: 10.1021/acsami.1c05938 (PMC8289230; doi:10.1021/acsami.1c05938)
Supplement: Supplementary file 1 — am1c05938_si_001.pdf [file am1c05938_si_001.pdf]

SUPPORTING INFORMATION

Interplay between electrolyte-gated

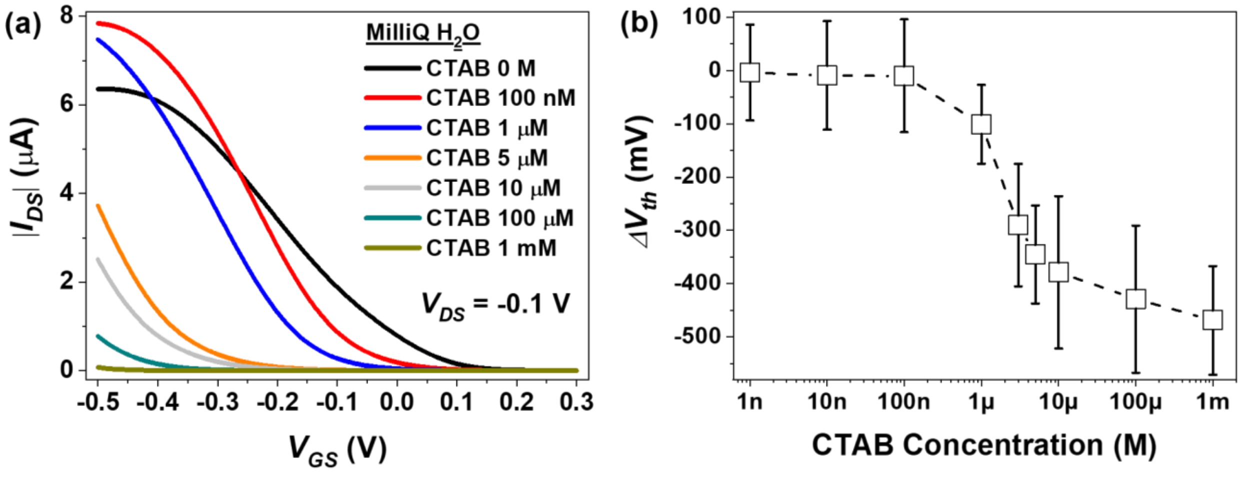

Figure S2. (a) Transfer characteristics of the same device displayed in Figure 2 recorded in linear regime. (b) ΔV_{th} versus CTAB concentrations in linear regime. The EGOFET device was exposed to a CTAB surfactant aqueous solution from 1 nM to 1 mM in ascending order.

Transfer characteristics of p-type EGOFETs using NaBr solution

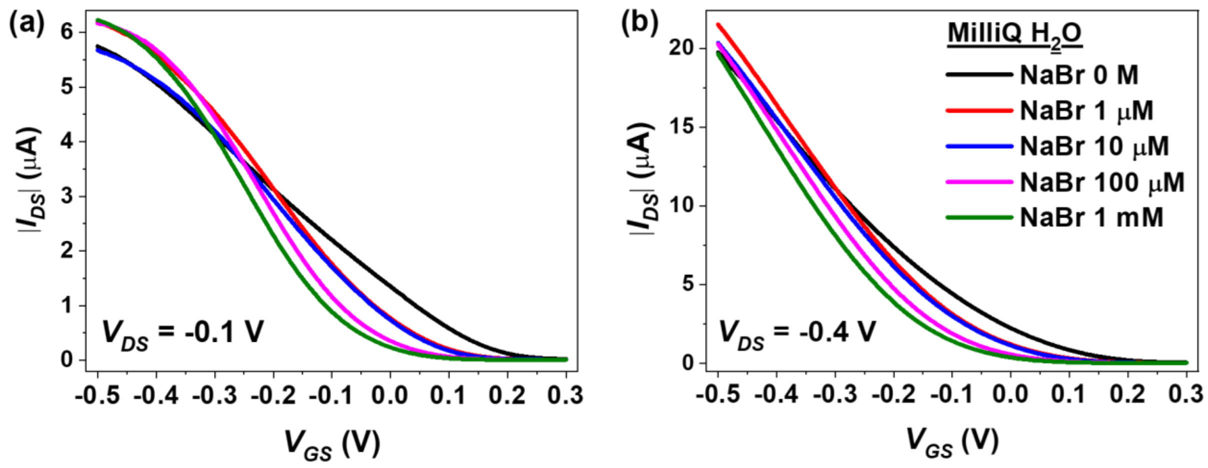

Figure S3. I-V transfer characteristics of a p-type EGOFET (based on diF-TES-ADT:PS blend) device recorded with different NaBr concentrations in MilliQ water as electrolyte media in (a) linear and (b) saturation regime.

Transfer characteristics of p-type EGOFETs with CTAB in PBS media

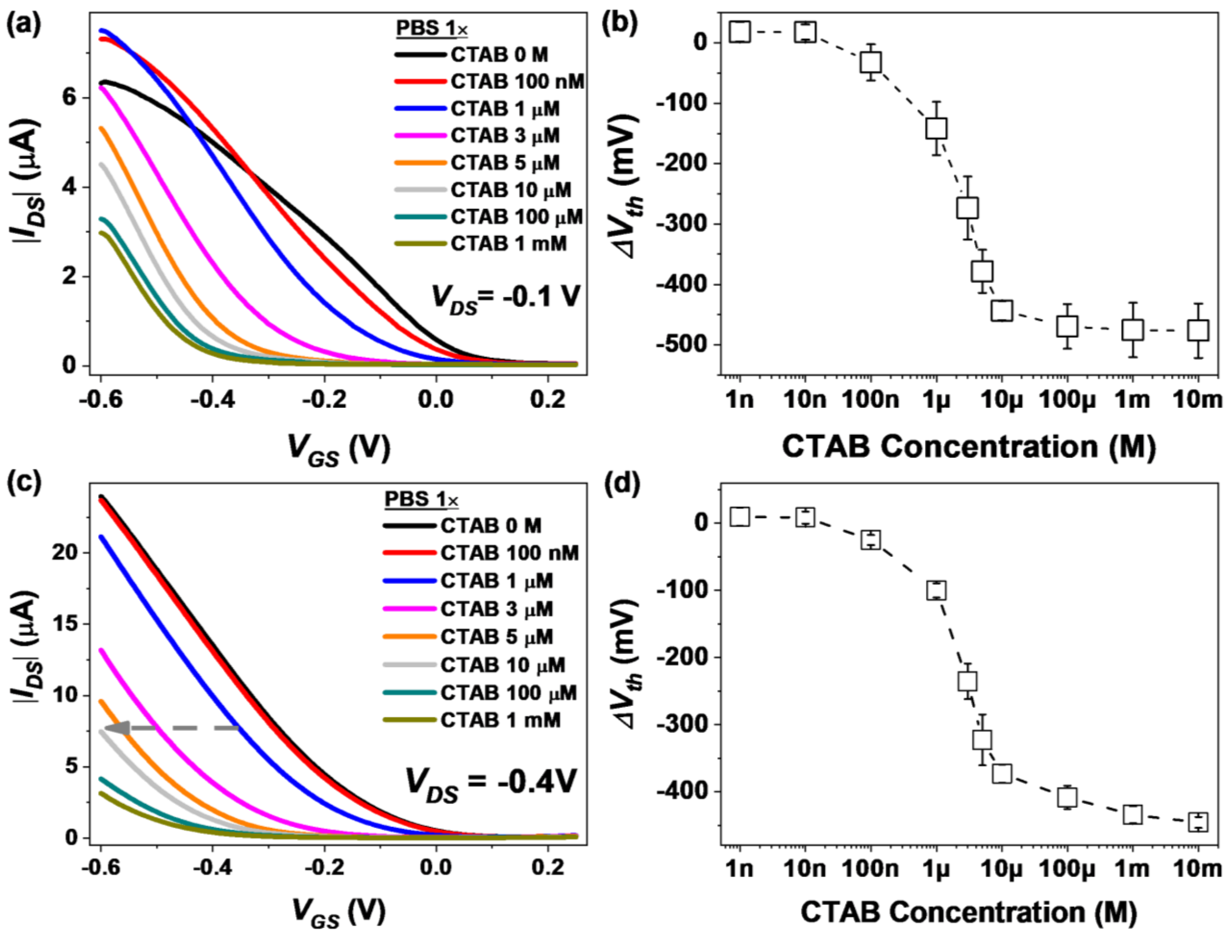

Figure S4. I-V transfer characteristics of p-type EGOFET (based on diF-TES-ADT:PS blend) using different CTAB concentration solutions (dissolving in PBS 1× as media) with Pt gate electrode recorded in (a) linear and (c) saturation regimes, respectively. Average threshold voltage shift ΔV_{th} plotted against different concentrations of CTAB solutions in (b) linear and (d) saturation regime, respectively. The EGOFET device was exposed to CTAB surfactant solutions from 1×10^{-9} M to 1×10^{-3} M in ascending order. These data were extracted from 4 different devices.

In order to clarify the orientation of the adsorption CTA^+ monolayer on charged Pt wire surface, the same transfer characteristics were repeated by using PBS 1× solution as solvent to dissolve CTAB surfactant. As mentioned in introduction, the high sensitivity of EGOFETs platform is mainly attributed to the EDL formation at OSC/electrolyte and gate

electrode/electrolyte interfaces. In EDL, the Debye screening length (λ_D) is a key parameter, which defines the effective detection length and strongly depends on the ionic strength of electrolyte.[1] In other terms, the adsorption of CTA^+ molecule on Pt wire is not expected to be detected if the charge of CTA^+ is beyond the λ_D. Typically, λ_D is ~ 100 nm in MilliQ water, while it is < 1 nm in PBS 1× solution.[2] Further, it is worth noting that the chain length of CTAB molecule is 2.3 nm.[3] Within this context, we would predict no apparent V_{th} shift in PBS 1× solution if a head-out adsorption of CTA^+ ions in the monolayer takes place (the head group of CTA^+ orientates to the solution). However, a similar negative V_{th} shift in PBS 1× solution would agree with a head-in adsorption of CTA^+ ions on the Pt surface.

As evidenced in Figure S4a and Figure S4c, a similar negative V_{th} shift is observed in PBS 1× solution with increasing CTAB concentration in linear regime and saturation regime, respectively. In addition, a pronounced negative V_{th} shift in the CTAB concentration range between 1×10^{-6} to 5×10^{-6} M is noticed in PBS 1× solution (dashed grey arrow line in Figure S4c). The relationship between ΔV_{th} and log[CTAB] in the PBS 1× solution in linear and saturation regime are plotted in Figure S4b and Figure S4d. Once more, the three regimes of adsorption are clearly distinguished.

Transfer characteristics of p-type EGOFET using a carbon nanocomposite gate electrode (carbon nanotubes)

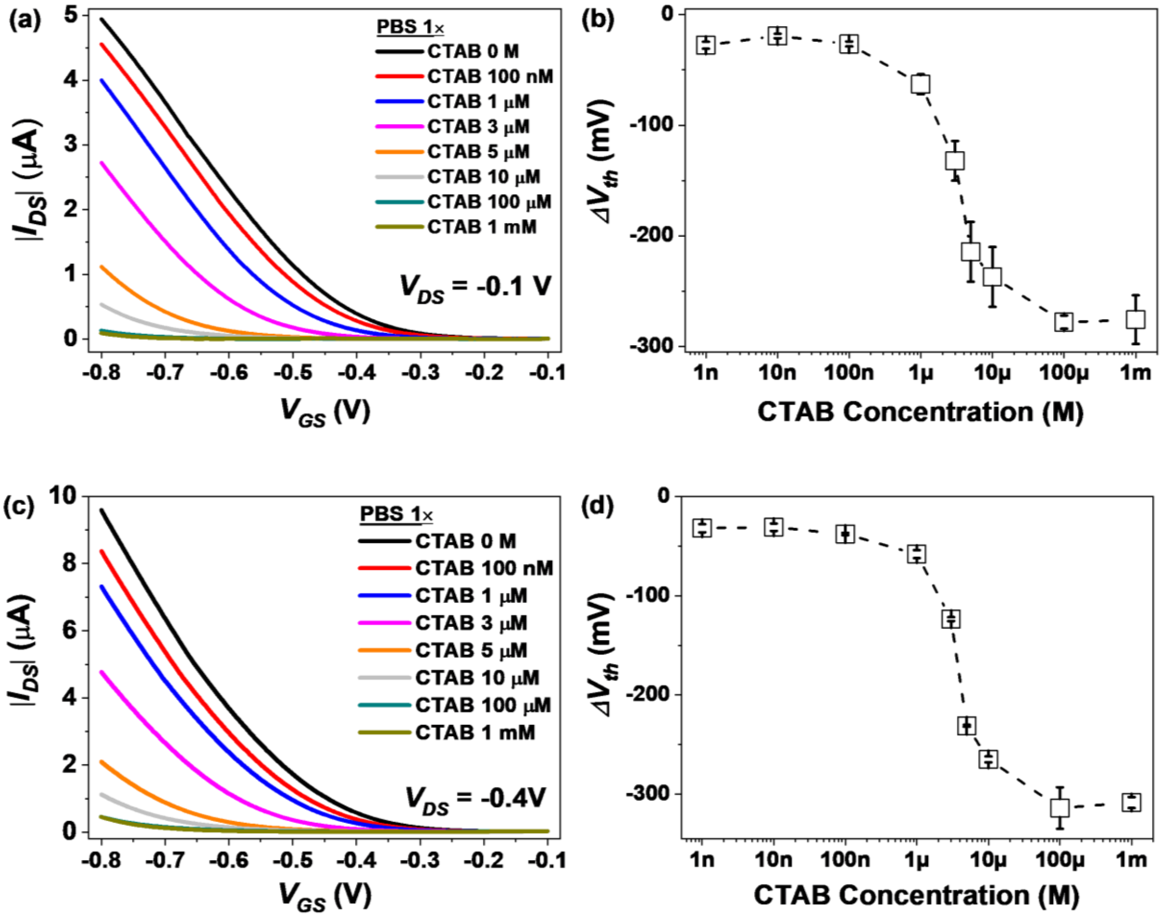

Figurer S5. I-V transfer characteristics of p-type EGOFETs using different concentrations of CTAB solutions (dissolved in PBS ×1 solution) with carbon paste electrode as gate electrode recorded in (a) linear and (c) saturation regime, respectively. Average threshold voltage shifting ΔV_{th} plotted against different concentrations of CTAB solutions (dissolved in PBS ×1 solution) in (b) linear and (d) saturation regime, respectively. These data were extracted from 4 devices.

CV and EIS results of CTA^{+} monolayer formation on Pt surface

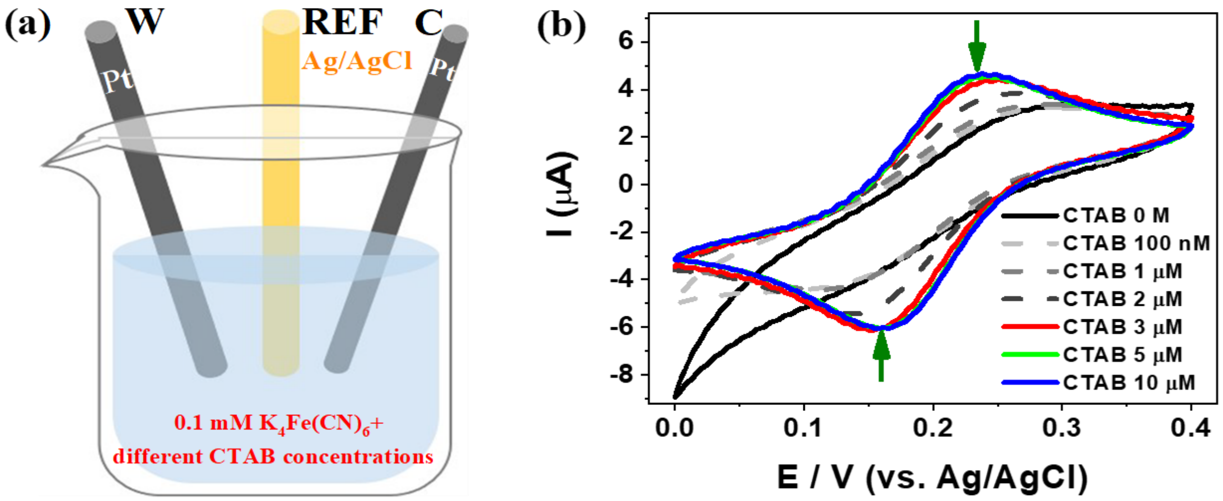

Figure S6. (a) Schematic vision of the cyclic voltammetry experiments. (b) Dependence of cyclic voltammetry for 0.1 mM $K_4Fe(CN)_6$ in 0.1 M KCl at the Pt electrode at different CTAB concentrations, i.e. 0, 100 nM, 10 0 0 QG D 0 6FDQ UDWH P9

Figure S6a displays the setup of the voltammetric response towards the redox probe $K_4Fe(CN)_6$ (1×10^{-4} M) to different CTAB concentrations. When CTAB concentration increases from 1×10^{-7} M to 2×10^{-6} M, the potential difference between the oxidation and reduction peaks (ΔE_p) gradual decreases and their peak current (I_p) is simultaneously increased (dashed lines in Figure S6b). However, with further increasing the CTAB concentration (from 3×10^{-6} M to 1×10^{-5} M, solid lines in Figure S6b), no changes of the ΔE_p and I_p in the voltammetric response are observed. The reduction of ΔE_p accompanied by the I_p enhancement related to $K_4Fe(CN)_6$ electronic charge transfer with the Pt working electrode, is attributed to the more favorable electrostatic interaction of the negatively charged redox probe with a positively charged monolayer of CTAB formed on the working electrode.[4] A similar response was observed in a similar cyclic voltammetry test performed by Hu et al.[4] Therefore, the above results agree with CTAB monolayer formation on the working electrode.

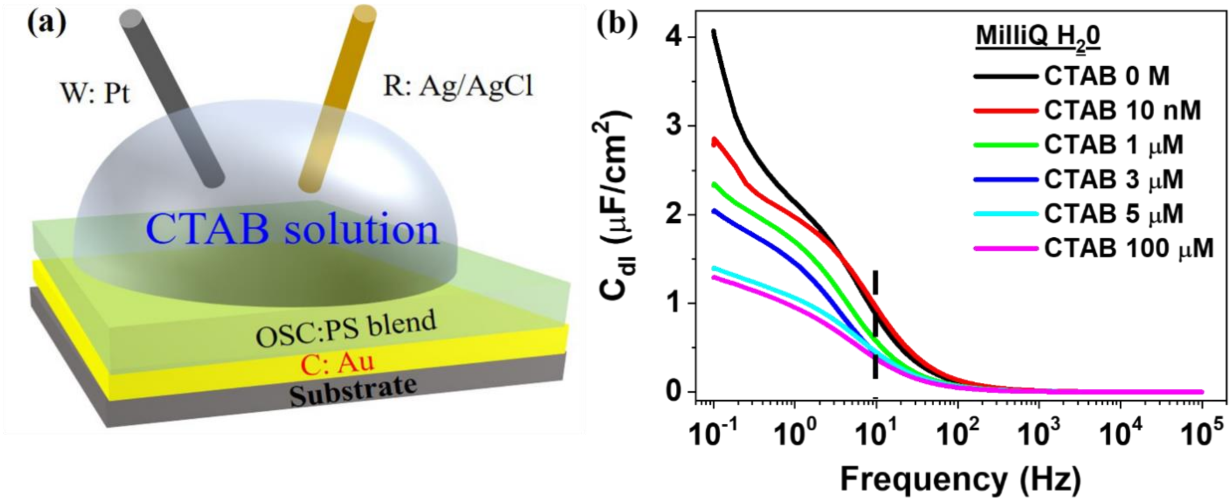

Figure S7. (a) The detection setup of EIS measurement. (b) Capacitance of Au platform recorded with different CTAB concentrations under V_{DC} = 0 V and V_{AC} = 20 mV. The CTAB is dissolved in MilliQ water at concentrations of 0, 10 nM, 1 M, 0 0 0 D Q G 0

The influence of CTAB monolayer on gate electrode was further investigated by electrochemical impedance spectroscopy (EIS) using the protocol described in Experimental Section. During EIS test, a Pt wire and a square Au electrode (S = 1 mm^2) were used as working and counter electrodes, respectively, as shown in Figure S7a. In addition, CTAB solution dissolved in MilliQ water with different concentrations were employed as media. Prior to each EIS measurement, a fast cyclic voltammetry (CV) scan was performed by applying a DC voltage in the same window used for transfer measurement (0.3 V to -0.5 V), aiming to promote the migration of CTA$^+$ ions towards the Pt wire. The EIS response shows a clear decrease of capacitance with CTAB concentration, as displayed in Figure S7b. For CTAB concentrations less than $1×10^{-8}$ M (red line), capacitance response does not change compared to the one U H F R U G H G L Q 0 L O O L ^{2} at 10 Hz). With increasing the surfactant concentration (from $1×10^{-8}$ M to $5×10^{-6}$ M), the capacitance value starts to drop (i.e.)^2 at 10 Hz, cyan line). With further increasing the CTAB concentration (> $5×10^{-6}$ M), a small decrease of the capacitance (i.e.)^2 at 10 Hz in $1×10^{-4}$ M CTAB solution, magenta line) is observed. Therefore, the formation of CTA$^+$ monolayer on Pt wire can be correlated to the sharp change of ΔV_{th} for transfer characteristics in presence of CTAB concentration less than $1×10^{-5}$ M.

Atomic Force Microscopy on a Au electrode after the growth of the CTAB surfactant monolayer

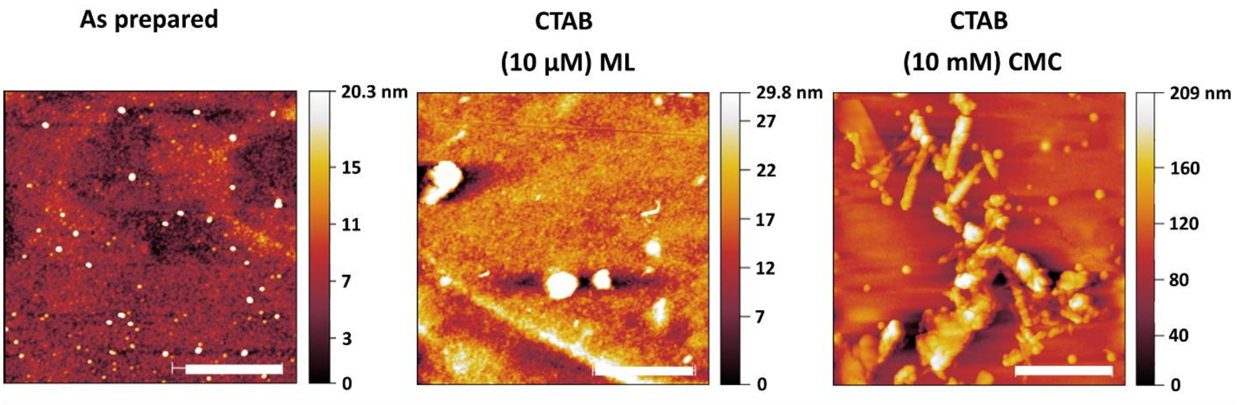

Figure S8. AFM image of Au gate electrodes on Kapton substrate. Each column represents a different condition of surfactant exposure: as prepared and after treating the substrate with CTAB at ML concentration and CMC concentration and applying a voltage sweep from 0.3 to -0.5 V. Scale bar 1 µm.

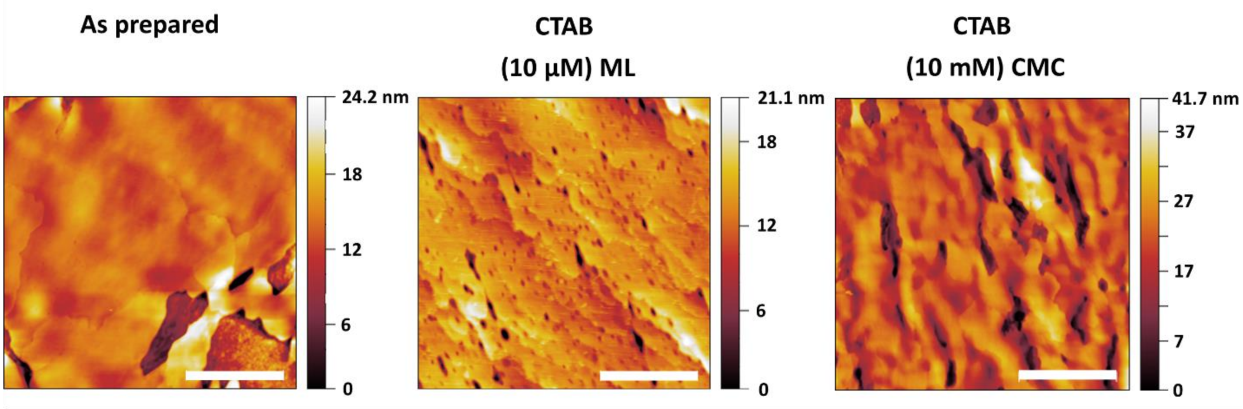

Figure S9. AFM image of diF-TES-ADT:PS blend film on Kapton substrate. Each column represents a different condition of surfactant exposure: as prepared, and after exposing the OSC surface to CTAB at ML and CMC concentration and applying a voltage sweep from -0.3 to 0.5. Scale bar 1 µm.

Transfer characteristics of p-type EGOFETs by incubating the film with the surfactant previously to performing the electrical test

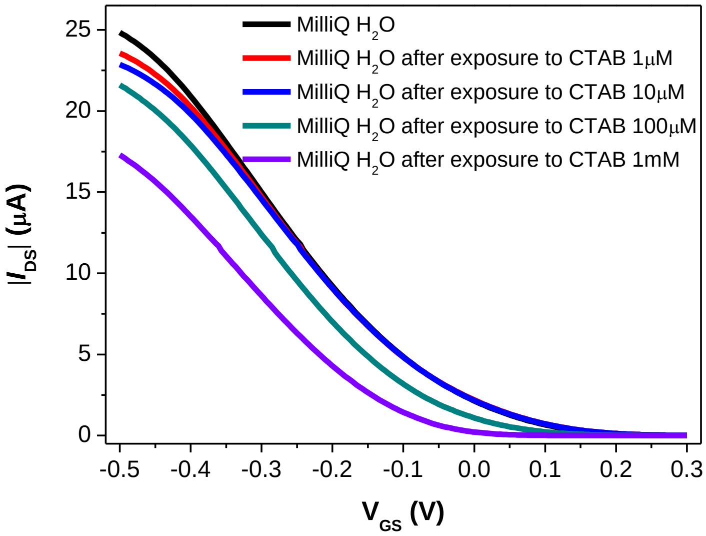

Figure S10. I-V transfer characteristics of p-type EGOFET recorded in saturation regime (V_{DS} = -0.4 V) in MilliQ water by exposure the OSC to different CTAB solutions (dissolving in MilliQ H_2O) followed by removing the CTAB solution and cleaning the Pt with MilliQ water three time.

Different CTAB solutions were casted on top of the OSC surface and kept for 3 mins followed by removing the CTAB solutions. Then, a droplet of MilliQ water was casted on the interdigitated area to measure the electrical response with a cleaned Pt wire. As displayed in Figure S10, only at concentrations above 100 µM some significant shifting of the curves is observed. Thus, although we observe some aggregation on the OSC above a certain concentration, this process is not sensitive at the monolayer formation concentration range. Further, it should be highlighted that this experiment is different from the one performed in Approach I, where the negatively polarization of the electrode will even disfavor more the aggregation of the surfactant on the OSC.

EIS results of DS monolayer formation on Pt surface

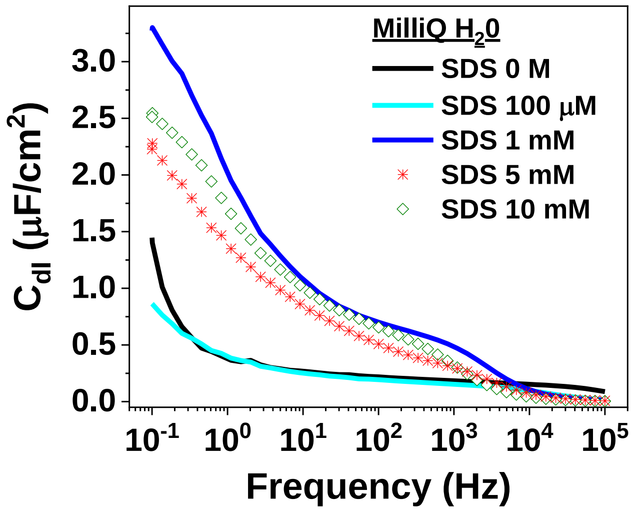

Figure S11. Capacitance measurements at different SDS concentrations Under V_{DC} = 0 V and V_{AC} = 20 mV. The SDS was dissolved in MilliQ water with different concentrations of 0, 100 0 1 mM, 5 mM and 10 mM.

To confirm the monolayer assembly of DS anions, EIS measurements were performed using the same setup as that used in CTAB experiments. Prior to capacitance recording, a fast CV measurement was performed in the same voltage window used in transfer test, aiming to push DS anions towards Pt electrode. As shown in Figure S8 (Supporting Information), firstly an increase of C_{dl} is observed with increasing the SDS concentration (blue line). At some point, a change of trend appears and a decrease of C_{dl} with increasing SDS concentration (red stars) is observed, followed by a gradual increase of C_{dl} with further increasing the SDS concentration (olive diamond). This trend is in alignment with our EGOFETs results

Transfer characteristics of n-type EGOFET using PBS 1× solution as solvent with SDS

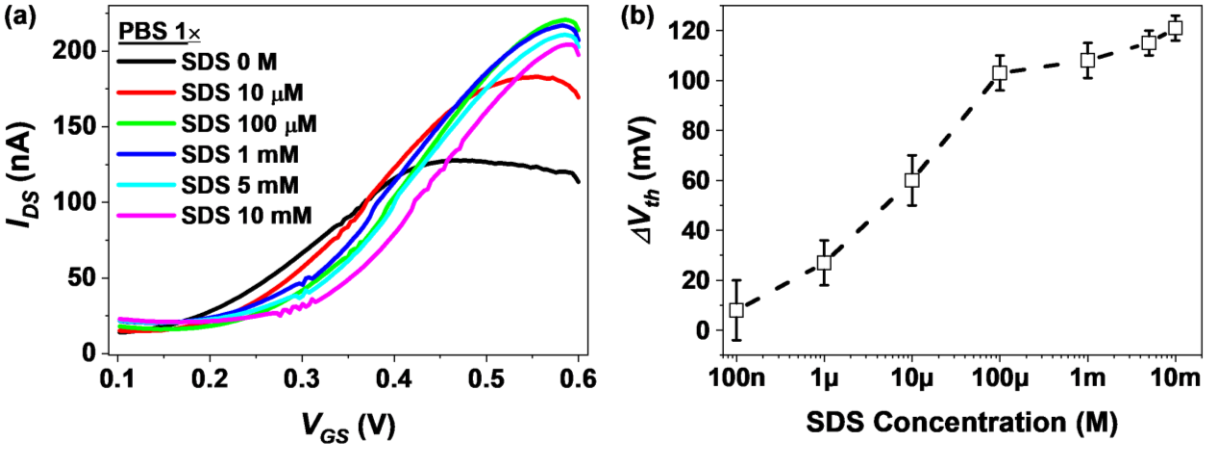

Figure S12. (a) I-V transfer characteristics of n-type EGOFETs (based on PDI8CN2:PS blend) using different concentrations of SDS solutions (dissolved in PBS×1 solution) with Pt as gate electrode recorded in saturation regime (V_{DS} = 0.5 V). (b) Average threshold voltage shift ΔV_{th} plotted against different concentrations of SDS solutions (dissolved in PBS×1 solution) in saturation regime. These data were extracted from 4 devices.

Atomic Force Microscopy of a Au electrode after the formation of the SDS monolayer

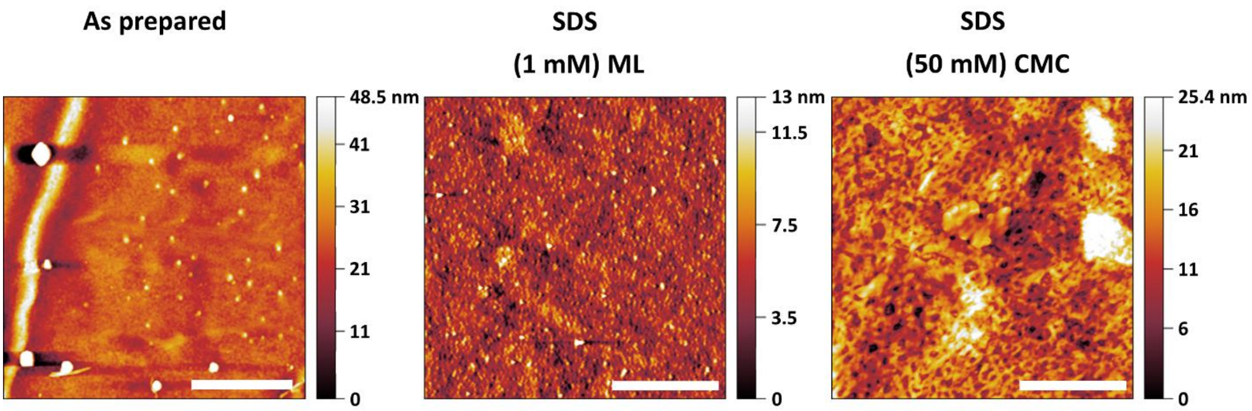

Figure S13. AFM image of a Au electrode on Kapton substrate. Each column represents a different condition of surfactant exposure: as prepared, and after exposing the electrode to SDS at ML and CMC concentration and applying a voltage sweep from -0.3 to 0.5. Scale bar 1 µm.

Determination of the optimized SDS concentration for the formation of a protective layer on top of OSC in p-type EGOFETs

To understand the optimized SDS concentration to form the protective layer on top of OSC in a p-type EGOFETs, different concentration of SDS solutions were tested. As displayed in Figure S12, the device cannot reach a stable electrical response in the absence of SDS (black curve) and after SDS 1 mM treatment (red curve). In contrast, despite an initial current drop during the first hour, a stable condition is reached in devices treated with 10 mM, 50 mM and 100 mM SDS solution, indicating the formation of a SDS protective layer on top of active OSC layer. Noticeably, all these three concentrations are above the CMC of SDS (8.2 mM at 298K in MilliQ water[5]), suggesting that the SDS concentration must lie above its CMC to form a complete protective layer. In addition, the *I/t* curve recorded in a device treated with 50 mM SDS solution, exhibits a lower I_{DS} decrease in the first operation hour as compared to the behavior observed in the case of the device treated with 100 mM SDS solution. According to our results, the optimized SDS concentration is 50 mM in MilliQ water.

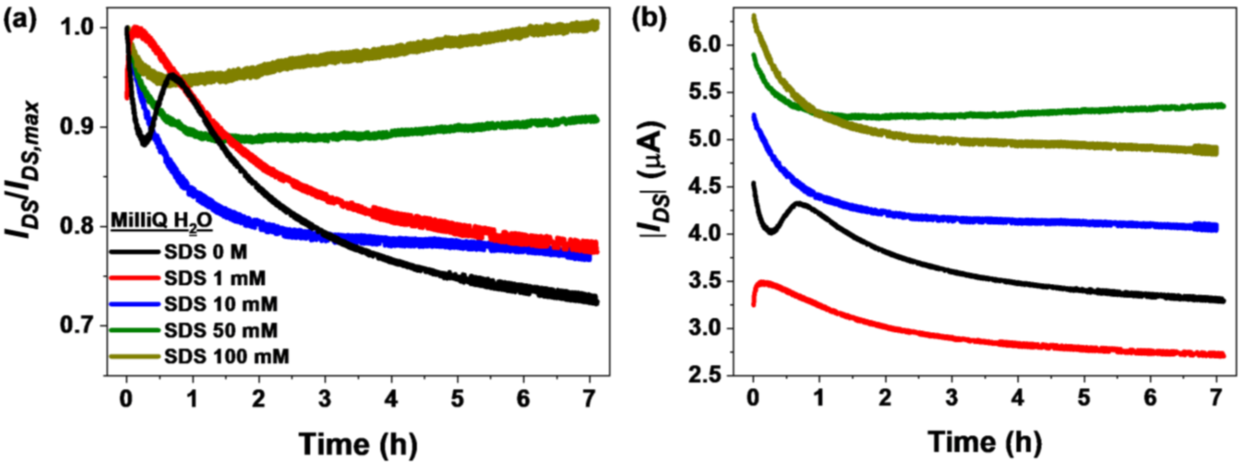

Figure S14. I-t plot of a p-type EGOFET (based on diF-TES-ADT:PS blend) (a) Normalized I_{DS} and (b) the measured I_{DS} recorded at V_{GS} = - 400 mV and V_{DS} = -50 mV with MilliQ water as media after treatment with different SDS concentrations.

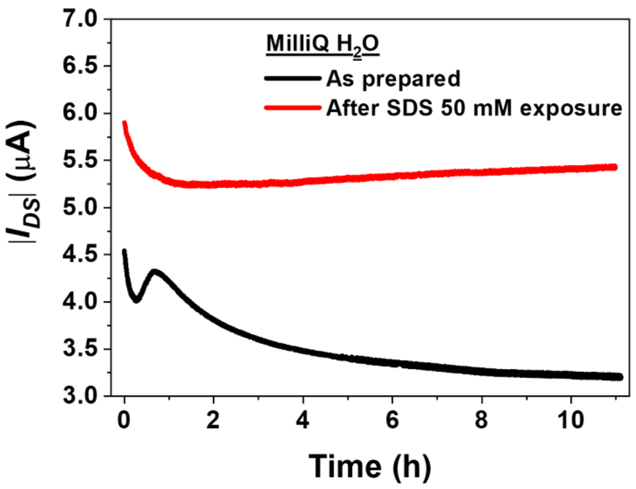

Figure S15: I-t plot of p-type EGOFETs (based on diF-TES-ADT:PS blend). I_{DS} was recorded at V_{GS} = - 400 mV and V_{DS} = -50 mV in the absence of SDS and after the treatment of the organic semiconductor with 50 mM SDS solution. All the data were recorded with MilliQ water as electrolyte.

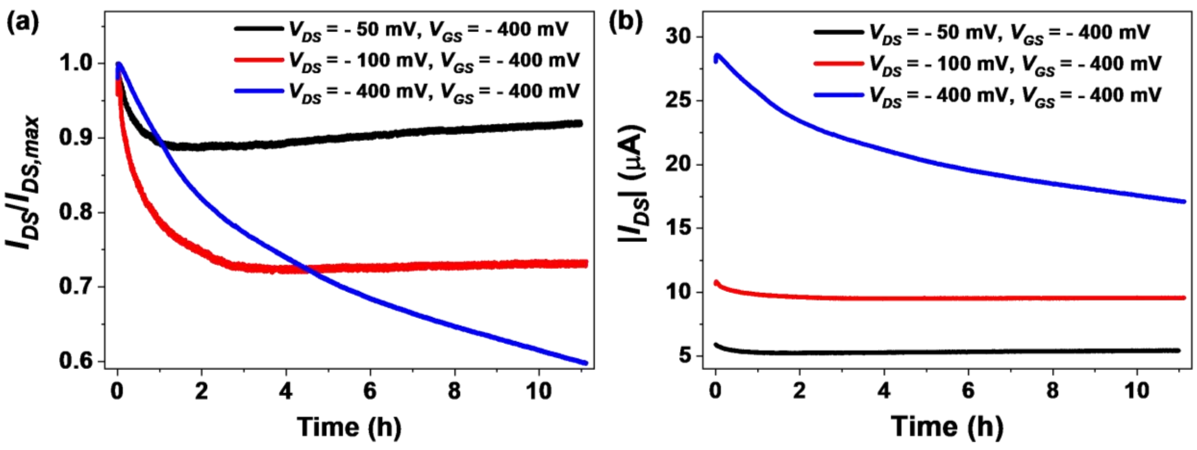

Figure S16. I-t plot of a p-type EGOFET (based on diF-TES-ADT:PS blend) after treating the organic semiconductor with 50 mM SDS. (a) Normalized I_{DS} and (b) measured I_{DS} data recorded at fixed V_{GS} = -400 mV but different V_{DS} voltages. The electrolyte medias was MilliQ.

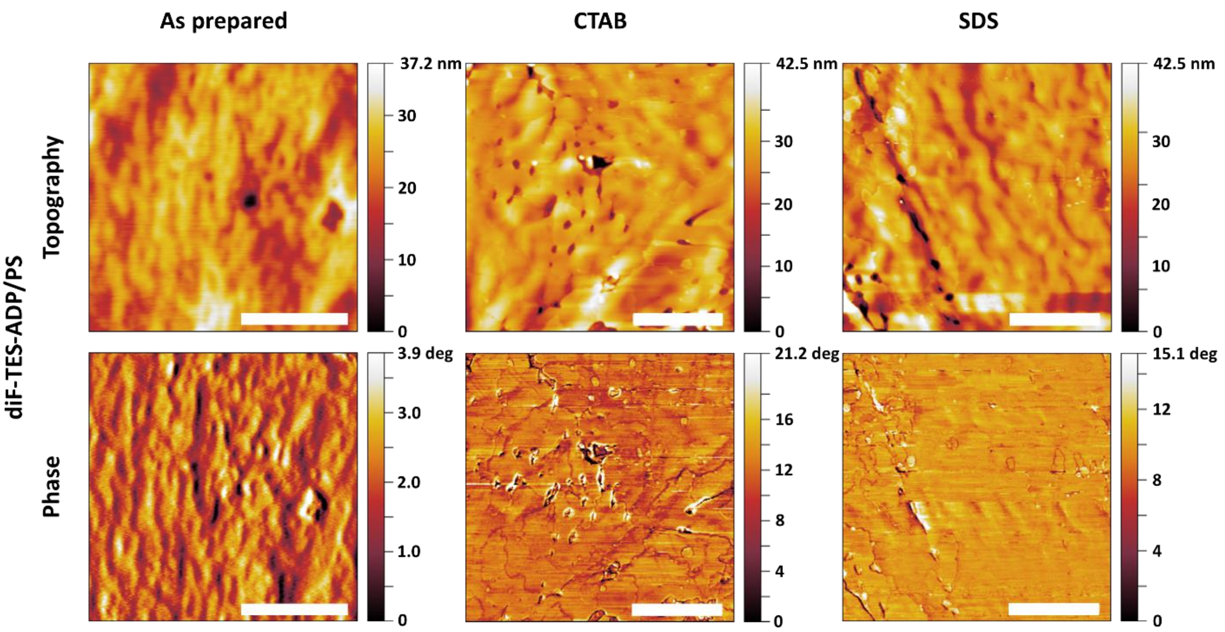

Figure S17. AFM images of diF-TES-ADT:PS blend film on Kapton for a fresh surface and after exposure to 10 mM CTAB and 50 mM SDS solution for 20 min. Scale bar 1 µm.

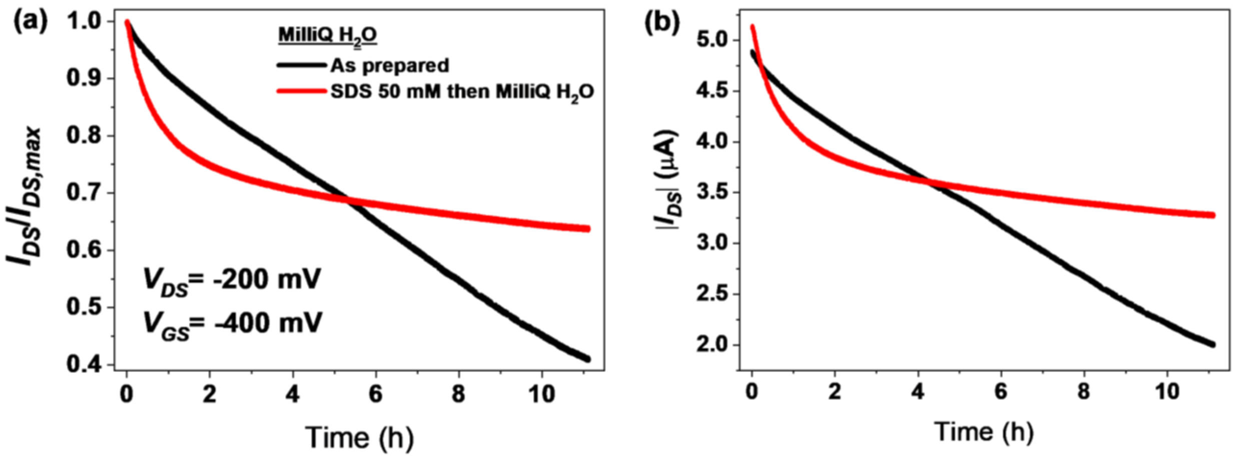

Figure S18. I-t plot of diF-TES-ADT-based EGOFET. (a) Normalized I_{DS} and (b) measured I_{DS} recorded at fixed operation voltage (V_{GS} = - 400 mV and V_{DS} = -200 mV) with MilliQ water as media in the absence of SDS (black curve) and after a treatment with 50 mM SDS solution (red curve). The pristine diF-TES-ADT film used in this device was deposited by coating diF-TES-ADT precursor (2 wt%) through the bar-assisted meniscus shearing (BAMS) technique at a fixed speed of 1 cm·s^{-1} and plate temperature of 105ºC in ambient conditions as previously reported.[6]

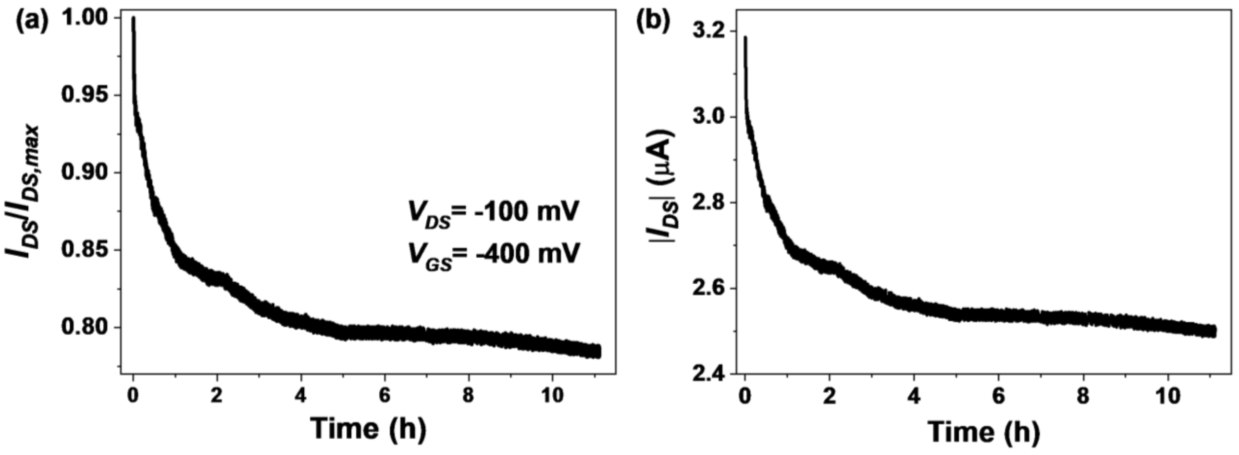

Figure S19. I-t plot of a p-type EGOFET (based on diF-TES-ADT:PS blend) after a treatment with 150 mM sodium octyl sulfate (SOS) solution. The CMC of SOS is 134 mM in MilliQ water at RT.[7] (a) Normalized I_{DS} and (b) measured I_{DS} recorded at fixed operation voltage (V_{GS} = - 400 mV and V_{DS} = -100 mV) with MilliQ water as media.

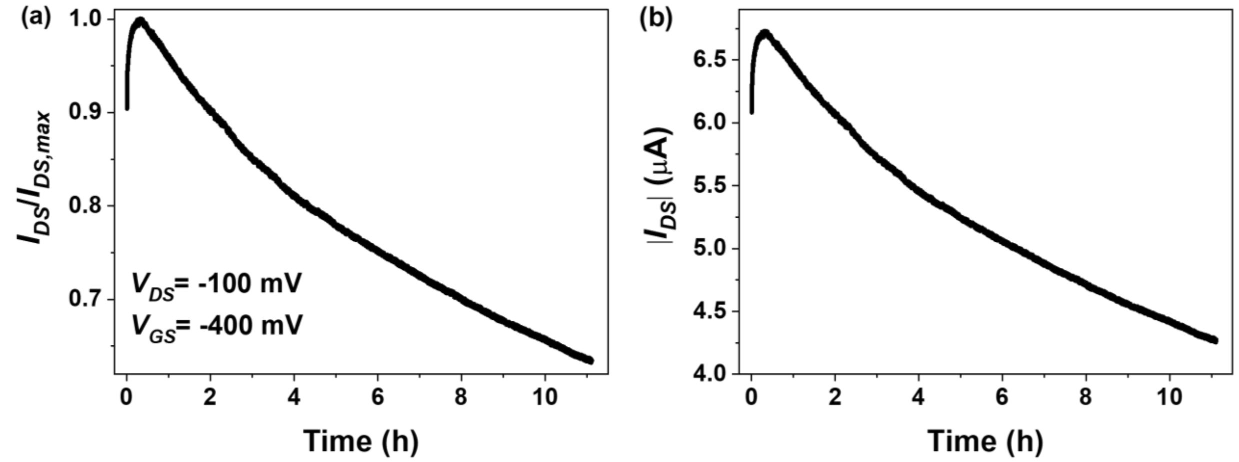

Figure S20: I-t plot of a p-type EGOFET (based on diF-TES-ADT:PS blend) after treatment with 10 mM CTAB solution. (a) Normalized I_{DS} and (b) measured I_{DS} recorded at fixed operation voltage (V_{GS} = - 400 mV and V_{DS} = -100 mV) with MilliQ water as media.

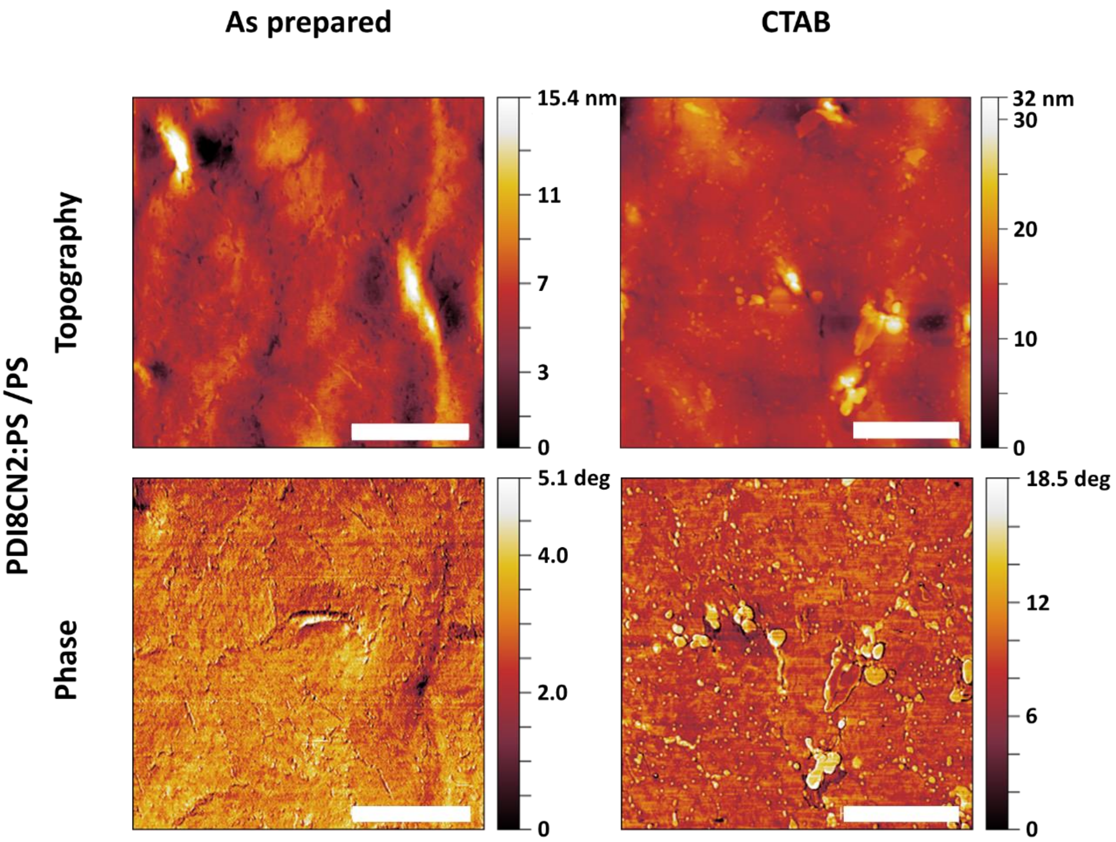

Figure S21: AFM image (5×5 m^2) of PDI8CN2:PS blend on Kapton substrate for a fresh film, and after exposure to CTAB 10 mM solution for 20 min.

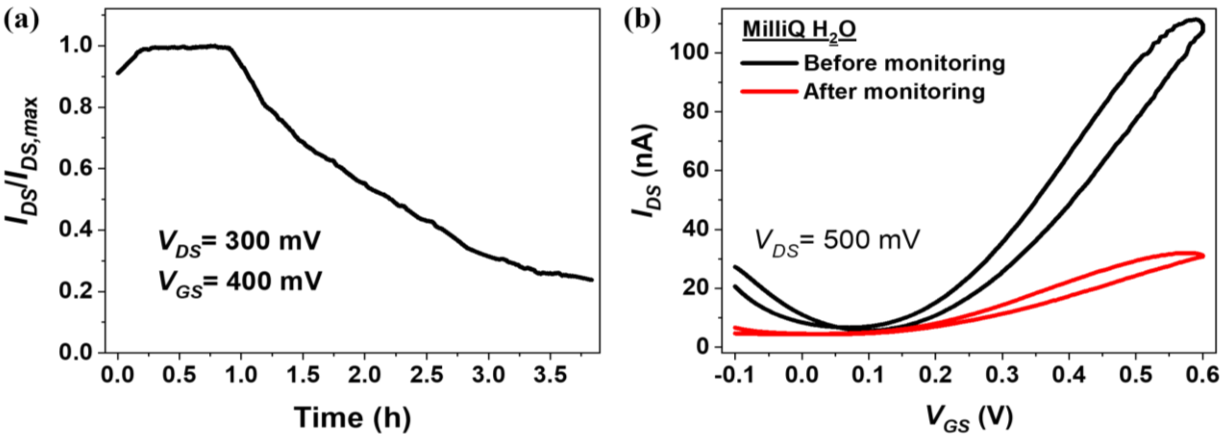

Figure S22. I-t plot of an n-type EGOFET (based on PDICN2:PS blend) (a) Normalized I_{DS} recorded at fixed operation voltage (V_{GS} = 400 mV and V_{DS} = 300 mV) with MilliQ water as media in the absence of CTAB treatment. (b) I-V transfer characteristics recorded for the n-type

device using MilliQ water as media. The black curve corresponds to the fresh device and the red one is the device response after performing the current monitoring test.

Reference:

(1) Brown, M. A.; Goel, A.; Abbas, Z. Effect of Electrolyte Concentration on the Stern Layer Thickness at a Charged Interface. Angew. Chemie - Int. Ed. 2016, 55 (11), 3790±3794.

(2) Kergoat, L.; Piro, B.; Berggren, M.; Pham, M. C.; Yassar, A.; Horowitz, G. DNA Detection with a Water-Gated Organic Field-Effect Transistor. Org. Electron. 2012, 13 (1), 1±6.

(3) Zhang, T.; Xu, G.; Puckette, J.; Blum, F. D. Effect of Silica on the Structure of Cetyltrimethylammonium Bromide. J. Phys. Chem. C 2012, 116 (21), 11626±11634.

(4) Hu, C.; Dang, X.; Hu, S. Studies on Adsorption of Cetyltrimethylammonium Bromide at Carbon Paste Electrode and the Enhancement Effect in Thyroxine Reduction by Voltammetry and Electrochemical Impedance Spectroscopy. J. Electroanal. Chem. 2004, 572 (1), 161±171.

(5) Häckl, K.; Li, H.; Aldous, I. M.; Tsui, T.; Kunz, W.; Abbott, A. P.; Warr, G. G.; Atkin, R. Potential Dependence of Surfactant Adsorption at the Graphite Electrode/Deep Eutectic Solvent Interface. J. Phys. Chem. Lett. 2019, 10 (18), 5331±5337.

(6) Zhang, Q.; Leonardi, F.; Casalini, S.; Temiño, I.; Mas-Torrent, M. High Performing Solution-Coated Electrolyte-Gated Organic Field-Effect Transistors for Aqueous Media Operation. Sci. Rep. 2016, 6, 39623.

(7) Ranganathan, R.; Tran, L.; Bales, B. L. Surfactant- and Salt-Induced Growth of Normal Sodium Alkyl Surfate Micelles Well above Their Critical Micelle Concentrations. J. Phys. Chem. B. 2000, 104, 2260-2264.
